# Interaction effects between possession status and percentage: insights from modeling match-running performance across possession status in male soccer

**DOI:** 10.5114/biolsport.2025.151653

**Published:** 2025-07-16

**Authors:** Pengyu Pan, Carlos Lago-Peñas, Miguel Lorenzo-Martinez, Robert Rein, Tianbiao Liu, Daniel Memmert, Ricardo Resta Serra, Roberto López del Campo

**Affiliations:** 1 Institute of Exercise Training and Sports Informatics, German Sport University Cologne, Cologne, Germany; 2 Faculty of Education and Sport Sciences, Universidade de Vigo, Spain; 3 College of Physical Education and Sport, Beijing Normal University, Beijing, China; 4 Department of Competitions and Mediacoah, LaLiga, Madrid, Spain

**Keywords:** Association football, Physical performance, Effective playing time, Ball possession, Linear mixed model

## Abstract

Due to the dynamic and complex nature of soccer, match-running performance (MRP) is highly influenced by match content. This study aimed to examine the interaction between possession status (PS) and possession percentage (PP) in relation to match-running performance (MRP) and to quantify MRP in each PS while considering multiple contextual variables. MRP indicators, including total distance (TD) and high-intensity running distance (HID), were collected from 8,468 observations of 412 outfield male players in the 2018–2019 Spanish LaLiga, excluding matches with red cards. This study set PS, possession percentage (PP), effective playing time, match location, quality of opposition, and match results as fixed effects, and set players and teams as random effects. Results indicated: i) PP interacted with PS, negatively affecting TD (r = -0.26, p < 0.05) and HID (r = -0.11, p < 0.05) during IP but positively influencing TD (r = 0.24, p < 0.05) and HID (r = 0.28, p < 0.05) during OP; ii) MRP during in-possession exceeded out-of-possession when PP was below 36% for TD and 36.4% for HID; iii) PP thresholds for MRP shifts varied by position, with forwards requiring higher PP (TD: 61.8%, HID: 68.6%) compared to central defenders (TD: 28.3%, HID: 9.2%). This study reveals the interaction effects of PS and PP on MRP, emphasizing the complexity of multivariate relationships in soccer. It underscores the importance of multivariate approaches over traditional methods like t-tests, which provide only partial insights.

## INTRODUCTION

Match-running performance (MRP), which is used to quantify players’ locomotor activities during match play, has been studied in recent decades due to their strong association with players’ physical and training status [1, 2]. Typical metrics of MRP such as total distance (TD) and high-intensity running distance (HID), were found to be closely associated with match outcome and team success [1, 2, 3]. Higher-ranked teams, for instance, are generally associated with better MRP, underscoring its importance in competitive contexts [1, 2, 4]. However, MRP is not a static measure but reflects different tactical purposes during different possession status (PS); it varies significantly depending on whether a team is in-possession (IP) or out-of-possession (OP) [5, 6, 7]. During IP phases, players must coordinate movements to create scoring opportunities, requiring both on-ball actions and off-the-ball runs. Conversely, OP phases demand rapid defensive organization, pressing, and spatial recovery to regain possession. Due to such significant differences in match context and tactical objectives between IP and OP, MRP is influenced by PS, reflecting the distinct physical demands associated with attacking and defending phases [6, 8, 9]. This duality raises a critical question: do players run more during IP or OP phases, and how does this depend on contextual factors such as possession percentage (PP)?

Previous studies generally reported higher MRP during OP compared with IP [4, 5, 10]. Additionally, possession percentage (PP) has been identified as a key factor influencing MRP. For instance, previous studies found that high-possession teams typically exhibited better MRP during IP, whereas low-possession teams engage in more high-intensity running distances during OP [11, 12, 13]. Furthermore, low-possession teams are expected to engage in more high-intensity running during the defending phase when facing higher-ranking opponents [13]. While some prior studies have incorporated PS, many traditional approaches analyze MRP using absolute match totals rather than normalized metrics. This may overlook the influence of PP, as a higher PP allows more time for IP and less time for OP.

To address these limitations, MRP can be evaluated on a per-minute basis, incorporating effective playing time (EPT)—a measure that accounts for the actual time the ball is in play. EPT in soccer is defined as the period during which the ball is in play and in possession of either team, excluding stoppages for fouls, injuries, substitutions, and other interruptions [14, 15], which can provide a more precise understanding of the physical demands in soccer matches [16, 17, 18]. Previous research demonstrated the impact of EPT on MRP, such as its influence on total distance and accelerations [16]. Additionally, contextual factors like PP and playing position significantly affect MRP. For instance, PP has opposite effects on MRP in IP and OP, with positional differences further shaping MRP [5, 10, 16]. Moreover, previous studies have confirmed the significant impact of match location [19, 20], quality of opposition [19, 20], and match results [21, 22] on match performance, highlighting the necessity of considering these contextual variables when analyzing MRP.

Despite these insights, existing studies have yet to comprehensively analyze MRP differences between IP and OP phases or integrate multiple contextual factors into their frameworks. Prior research relies on traditional statistical methods, such as t-tests, which oversimplify the complex interactions between PS and PP [10, 11]. For instance, studies which used t-tests to compare MRP between IP and OP in isolation, suggested teams are supposed to perform greater MRP during OP than IP [10, 11]. Those results ignored how PP thresholds—specific values where MRP dominance shifts—modulate these relationships. This methodological limitation hinders a nuanced understanding of MRP and its positional variations, particularly in elite soccer contexts.

To address these gaps, this study employs a linear mixed-model framework to investigate two primary research questions:

1. *Which PS (IP or OP) requires greater MRP (measured in TD · min^-1^ and HID · min^-1^) in soccer matches, and how does this depend on PP thresholds?*
2. *How do analysis results differ between traditional t-tests and models incorporating multiple contextual variables?*

Therefore, this study aimed to first investigate the interaction effect of PP and PS on MRP, and then used linear mixed modes with considering EPT, PS, and other contextual factors to quantify MRP during each PS.

## MATERIALS AND METHODS

### Participants

The dataset of this study was from the Spanish professional male soccer league, LaLiga, during the 2018–2019 season. Since dismissals would influence the numerical balance, requiring the team with a dismissed player to compensate with greater individual running demands [23], matches that included red cards were excluded. In addition, referring to similar research in LaLiga [18], the individual data were selected only from the outfield players (goalkeepers were excluded) who completed the entire match. Hence, the last dataset comprised 8,468 individual match observations of 412 outfield players from 20 teams. Additionally, we classified players into five positions based on previous literature [18, 24]: central defenders (CD), external defenders (ED), central midfielders (CM), external midfielders (EM), and forwards (F). Data were obtained from the Spanish Professional Football League (LaLiga), permitting the use of the variables included in this study. To protect the privacy of players and teams, all data were anonymized in accordance with the principles of the Helsinki Declaration, and the investigation was approved by the local ethics committee (application number: 093/2017).

### Variables and Procedure

Regarding to the MRP indicators, the present study considered the total distance (TD), and high-intensity running distance (HID, > 21.0 km/h) covered by individual players. MRP was tracked by a multicamera computerised optical tracking system TRACAB^®^ (Chyronhego, New York, USA), and managed from Mediacoach^®^ software (LFP, Madrid, Spain) to monitor match activities. This multiple-camera system passively tracks the movements of every player using a sampling rate of 25.0 Hz over the entire course of matches, and the validity and reliability of the system have been described and verified, with reported positional and speed errors of 0.08 m and 0.5% respectively [25, 26].

### Statistical analysis

**FIG. 1:**
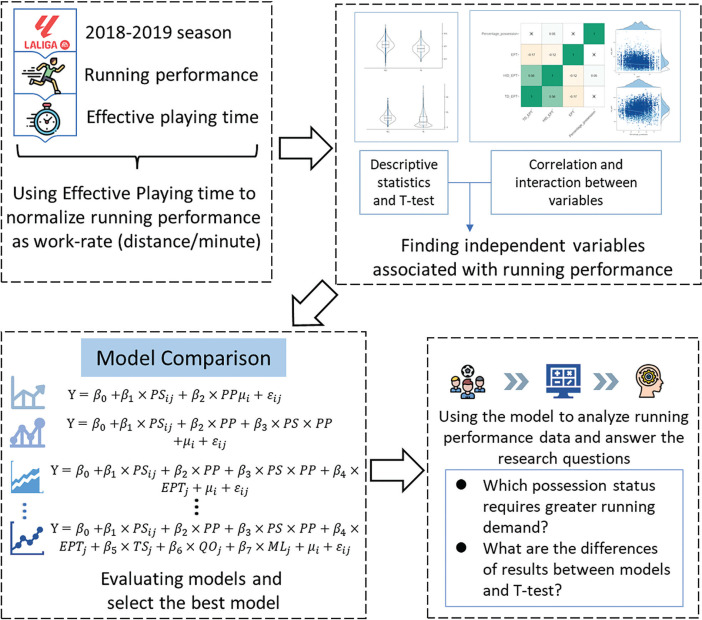
Study design.

Regarding analyzing process of this study, all the steps were demonstrated in Figure 1.

This study first calculated the relative MRP during different PS, making all running performance normalized to meters per unit of time (m/min) according EPT, and then conducted descriptive analysis using t-test and Cohen’s d effect size to identify statistical difference. Effect size was calculated and interpreted as follows: 0.00–0.19: trivial; 0.20–0.59: small; 0.60–1.19: moderate; 1.20–1.99: large; ≥ 2.00: very large [27]. For all analyses, the significance value was set at *p* < 0.05. All data was imported into R Statistical Software (ver.4.1.1; R Core Team 2022) for pre-processing and statistical analysis.

And then this study used *Pearson’s* correlation coefficient to measure the linear correlation between the running performance indicator and several influential factors. Regarding the construction of linear mixed models, although various factors may influence MRP, we selected key contextual factors based on previous literature [5, 8, 9, 16, 19, 20]. Finally, we set PS, PP, EPT, match location (ML), quality of opposition (QO), and match results (MR) as independent variables on MRP. To investigate the interaction between PS and PP, we conducted the correlation test respectively for IP and OP to check the interaction between PS and some other variables. Then linear mixed models were built using package “LmerTest” [28], and we constructed several models step by step with from using only PS to all the variables. The assumptions of homogeneity and normality of the residuals were verified for each model, with no specific issues identified.

Regarding model evaluation, this study used the marginal *R^2^*, and conditional *R^2^* to provide an absolute value for the goodness-of-fit of a model [29, 30]. The marginal *R^2^* was used to evaluate the fixed effect of the model, and the conditional *R^2^* was used to calculate the overall *R^2^* of fixed effect and random effect [30].

After the model evaluation, the final linear mixed model was constructed as follows:

$$\begin{array}{l} \begin{array}{l} MRP_{ij}=\beta_{0}+\beta_{1}\times PS_{ij}+\beta_{2}\times PP_{ij}\\ \;+\beta_{3}\times PS_{ij}\times PP_{ij}+\beta_{4}\times EPT_{ij}\\ \;+\beta_{5}\times QO_{ij}+\beta_{6}\times ML_{ij}+\beta_{7}\times MR_{ij} \end{array}\}fixed\\ \;+\mu_{i}+\mu_{j}+\varepsilon_{ij}\;\rangle \;random \end{array}$$ (1)

The general specification of model (1) for an individual player observation’s match running performance, *MRP*_ij_ on player *i* within the *j*-th team is shown in formula (1). In this model, fixed effect included PS, PP, an interaction of PS and PP, EPT, QO, ML, and MR. The random effect associated with the intercept for team *j* and player *i* is indicated by μ_j_ and μ_i_. We write the distribution of these random effects as:

$$\mu_{j}∼𝒩(0,\sigma_{team}^{2}),\text{ and }\mu_{i}∼𝒩(0,\sigma_{player}^{2})$$

where $\sigma_{team}^{2}$ and $\sigma_{player}^{2}$ represent the variance of the random team effects and player effects respectively.

And the distribution of the residual *ɛ_ij_*, associated with the observation on an individual player *i* within team *j*, is described as following:

$$\varepsilon_{ij}∼𝒩(0,\sigma^{2})$$

## RESULTS

Table 1 showed the result of descriptive statistic. Regarding to all players, the results showed MRP during IP is lower than OP (*p* < 0.001). Moreover, players who played as CD, ED, and CM showed similar results of MRP during different PS, as higher TD (*p* < 0.001), and HID (*p* < 0.001) during IP than OP. The normalised TD during IP of EM is lower than OP (*p* = 0.002), while the results of HID are different as MRP in IP is greater than OP (*p* < 0.001). Additionally, MRP of F during IP is higher than OP including both TD (*p* < 0.001) and HID (*p* < 0.001).

**TABLE 1:** Descriptive statistics of running performance between in-possession and out-of-possession (Mean ± SD)

	MRP (m/min)	IP		OP		*p*	ES

		Mean ± SD	95% CI	Mean ± SD	95% CI		
In total	TD	141.29 ± 19.04	140.71, 141.86	157.19 ± 19.94	156.59, 157.79	< 0.001	-0.82
	HID	9.15 ± 7.83	8.91, 9.38	12.74 ± 5.41	12.58, 12.91	< 0.001	-0.53

CD	TD	124.87 ± 12.72	124.16, 125.59	151.48 ± 15.53	150.61, 152.35	< 0.001	-1.87
	HID	2.6 ± 1.91	2.49, 2.71	13.25 ± 5.11	12.96, 13.53	< 0.001	-2.76

ED	TD	140 ± 13.6	139.11, 140.88	156.89 ± 14.46	155.95, 157.83	< 0.001	-1.20
	HID	11.93 ± 5.38	11.58, 12.28	15.07 ± 5.38	14.73, 15.42	< 0.001	-0.59

CM	TD	150.58 ± 15.24	149.64, 151.52	174.18 ± 16.33	173.17, 175.19	< 0.001	-1.49
	HID	5.92 ± 4.65	5.64, 6.21	12.77 ± 5.31	12.44, 13.09	< 0.001	-1.37

EM	TD	155.3 ± 17.18	153.8, 156.79	158.91 ± 19.61	157.21, 160.61	0.002	-0.20
	HID	17.08 ± 7.5	16.43, 17.73	12.14 ± 4.98	11.71, 12.57	< 0.001	0.78

F	TD	149.59 ± 19.77	147.97, 151.2	138.55 ± 18.62	137.03, 140.07	< 0.001	0.57
	HID	17.33 ± 8.05	16.67, 17.99	8.46 ± 3.83	8.15, 8.78	< 0.001	1.41

Abbreviations: MRP match-running performance; CI confidence interval; ES effect size; TD total distance; HID high-intensity running distance; IP in-possession; OP out-of-possession; CD central defenders; ED external defenders; CM central midfielders; ED external midfielders; F forwards

**FIG. 2:**
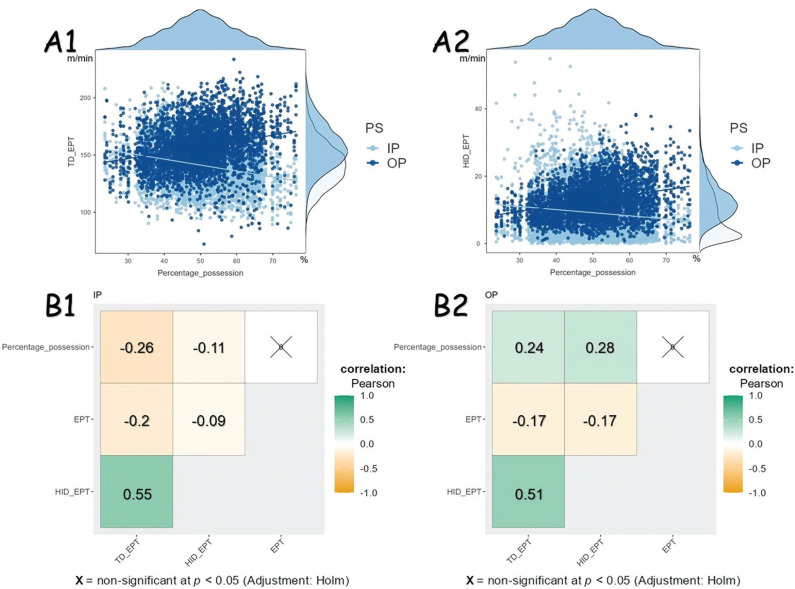
The interaction effect between possession status and possession percentage. Abbreviations: TD_EPT Total distance normalized by effective playing time; HID_EPT high-intensity running distance normalized by effective playing time; PS possession status; IP inpossession; OP out-of-possession; EPT effective playing time

Figure 2 shows the results of Pearson’s correlation test before building linear mixed-effect models. According to Figure 2(A1) and (A2), the results demonstrated that there is an interaction between PS and PP for both TD and HID. When the team is IP, PP could affect running performance negatively, but positively during OP.

Figure 2(B1) and (B2) showed the correlation test result with considering different PS, demonstrating that MRP is significantly correlated with percentage of possession (PP) and EPT. When team is IP, a significant negative correlation of PP was found between TD (*r* = -0.26, *p* < 0.05), and HID (*r* = -0.11, *p* < 0.05). In addition, when the team is OP, a significant positive correlation of PP was found between TD (*r* = 0.24, *p* < 0.05) and HID (*r* = 0.28, *p* < 0.05).

Table 2 demonstrated the evaluation results of different linear mixed models. The purpose of Model 1 to Model 3 is to evaluate the random effects in the model, as shown, setting both *Team* and *Player* as random effects is the best. Since the purpose is to explore which PS requires greater running performance, the PS is the main variable in the model. In addition, as we found in Figure 2, we compared the model with the interaction between PS and PP (Conditional *R^2^*: 0.567, Marginal *R^2^*: 0.201) with the model without interaction (Conditional *R^2^*: 0.510, Marginal *R^2^*: 0.148), and the result confirmed the interaction. Moreover, Model 10 to Model 17 demonstrated the addition of different independent variables to the model step by step.

**TABLE 2:** The results of model evaluation (dependent variable: total distance)

	Model		Conditional *R*^2^	Marginal *R*^2^

	Fixed effect	Random effect		
1	PS	Team	0.174	0.143
2	PS	Player	0.495	0.144
3	PS	Team, Player	0.496	0.143
4	PS + PP	Team, Player	0.510	0.148
5	PS^*^PP	Team, Player	0.567	0.201
6	PS^*^PP + EPT	Team, Player	0.575	0.225
7	PS^*^PP + QO	Team, Player	0.574	0.203
8	PS^*^PP + ML	Team, Player	0.567	0.201
9	PS^*^PP + MR	Team, Player	0.568	0.202
10	PS^*^PP + EPT + QO	Team, Player	0.575	0.225
11	PS^*^PP + EPT + ML	Team, Player	0.575	0.225
12	PS^*^PP + EPT + MR	Team, Player	0.575	0.225
13	PS^*^PP + QO + ML	Team, Player	0.574	0.203
14	PS^*^PP + QO + MR	Team, Player	0.574	0.203
15	PS^*^PP + ML + MR	Team, Player	0.569	0.202
16	PS^*^PP + QO + ML + MR	Team, Player	0.575	0.203
17	PS^*^PP + EPT + QO + ML + MR	Team, Player	0.576	0.225

* represents that there is an interaction between the two variables. Abbreviations: PS possession status; PP possession percentage; EPT effective playing time; QO quality of opposition; ML match location; MR match results

Table 3 shows the results of the final model. The results reflected that MRP during IP was greater than OP in general while MRP during OP would rise as PP increases. In addition, when the PP in the match is over 36%, and 36.4%, TD and HID during OP would be greater than IP, respectively.

**TABLE 3:** The results of the final model

		TD			HID		

		Beta	95% CI	*p*	Beta	95% CI	*p*
PS	IP	Baseline			Baseline		
	OP	-36	-39, -32	< 0.001	-9.1	-10, -7.8	< 0.001

ML	Away	Baseline			Baseline		
	Home	-0.08	-0.57, 0.72	-0.8	-0.39	-0.65, -0.13	-0.003

MR	Drawing	Baseline			Baseline		
	Losing	-0.66	-1.4, 0.12	-0.1	-0.06	-0.37, 0.26	-0.7
	Winning	-0.88	-1.7, -0.10	-0.028	-0.15	-0.47, 0.17	-0.4

PS * PP	IP * PP	Baseline			Baseline		
	OP * PP	-1	-0.97, 1.1	< 0.001	-0.25	-0.23, 0.28	< 0.001

PP		-0.43	-0.48, -0.37	< 0.001	-0.1	-0.12, -0.08	< 0.001
QO		-0.05	-0.11, 0.01	-0.1	-0.01	-0.03, 0.01	-0.5
EPT		-0.69	-0.77, -0.61	< 0.001	-0.17	-0.20, -0.14	< 0.001

Abbreviations; TD total distance; HID high-intensity running distance; CI confidence interval; PS possession status; IP in-possession; OP out-of-possession; ML match location; MR match result; PP possession percentage; QO quality of opposition; EPT effective playing time

Regarding other independent variables, ML affects HID significantly as MRP in home-matches would be lower than away-matches (Beta = -0.39, *p* = 0.003). As for the MR in the model, winning matches could show higher TD (Beta = -0.88, *p* = 0.028) compared to the drawing matches. In addition, PP (Beta = -0.43, *p* < 0.001) and EPT (Beta = -0.69, *p* < 0.001), which are continuous variables, both affected MRP negatively.

**FIG. 3:**
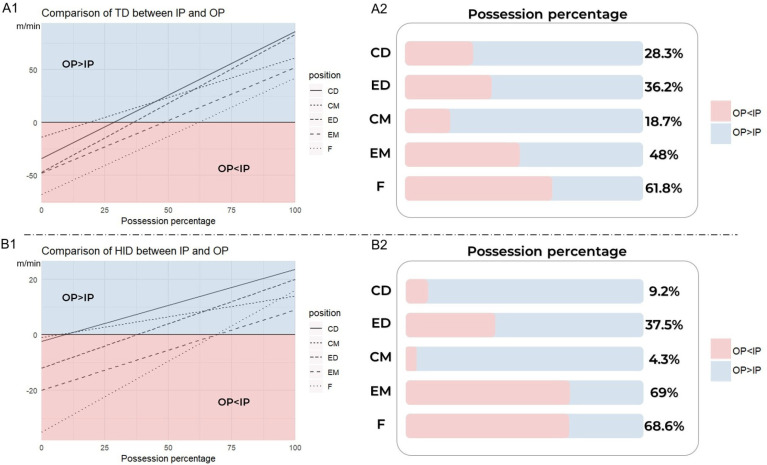
Model results according to different positions. The red part represents that the running performance during IP is greater than OP, and while blue part represents that the running performance during OP is greater than IP. Abbreviations: IP in-possession; OP out-of-possession; OP < IP based on specific possession percentage, the running demands in OP are lower than IP; OP > IP based on specific possession percentage, the running demands in OP are higher than IP; CD central defenders; ED external defenders; CM central midfielders; EM external midfielders; F forwards

Figure 3 shows the model results for the different playing positions. Regarding TD, Figure 3(A1) indicates that MRP during different PS would change according to possession percentage, and Figure 3(A2) depicts the specific value of PP for different positions when TD would be the same during IP and OP. For most positions, players’ TD during OP are greater than IP when PP is over 48%. And only the TD of F during IP is greater than OP when the PP is below 61.8%.

Moreover, Figure 3 (B1) and (B2) demonstrated the results of HID for different playing positions, which showed a similar changing trend of MRP according to PP with TD. The HID of players during OP would be higher than IP when PP is over 37.5% for CD, ED, and CM. However, when PP is almost over 70%, the running performance of HID during OP would be greater than IP for EM (69%) and F(68.6%).

**FIG. 4:**
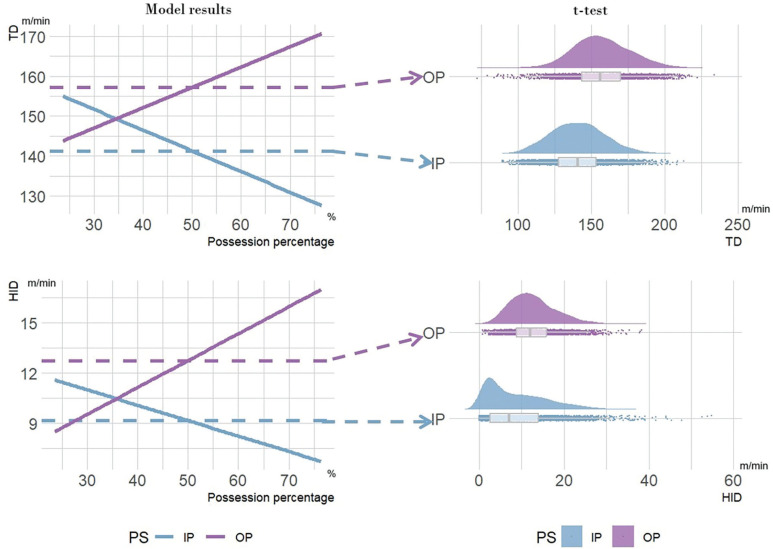
Comparison between t-test results and linear mixed models. The dotted lines represent the t-test results for running demands between in-possession (IP) and out-of-possession (OP). Abbreviations: TD: Total distance covered; HID: High-intensity running distance; PS possession status

The differences between the t-test and linear mixed model results are illustrated in Figure 4. This figure shows that the MRP between IP and OP are dynamically associated with the PP. Specifically, TD and HID during IP can be higher than during OP when PP is below approximately 35%. In contrast, the t-test results are static and indicate that TD and HID during IP are generally lower than during OP across all conditions.

## DISCUSSION

This study used multiple linear mixed models to explore players’ MRP during each PS, and mainly aimed to answer two specific research questions: (1) Which PS (IP or OP) requires greater MRP in soccer matches? (2) How do analysis results differ between t-tests and models incorporating multiple variables?

### MRP during IP and OP

In general, the results indicated that the MRP during each PS would change according to PP, as TD and HID during IP could be greater than OP when PP is lower than 36% and 36.4% respectively. While previous literature suggested that MRP during OP are higher than during IP [4, 5, 10], those previous findings could be one part of the results in this study. When PP is over the specific value, our results suggest similar to MRP during OP is higher than IP. Moreover, the observed phenomenon might be influenced by the duration of ball possession during matches. Previous studies have primarily compared MRP using absolute distances covered during matches [4, 11, 13]. However, since teams can have different ball possession strategies, the time spent on IP or OP also changes accordingly. For instance, teams with higher PP typically have more time on possessing the ball and less time for defending. Therefore, it is the reality that teams with high PP have better MRP during IP than teams with lower PP [11, 13]. As for directly comparing running performance across matches may lead to inaccuracies [17, 18], this is the reason why this study addressed this issue by using normalised MRP (m/min) to evaluate match performance.

Moreover, several related studies have also considered EPT to calculate MRP, which revealed significant correlations between MRP, EPT, PP, and match outcomes [13, 16, 17, 18]. Their findings aligned with this study, as we found that EPT affected MRP negatively. Since EPT is linked to match intensity and stoppages [14, 15], more EPT represents higher intensity and fewer stoppages. Consequently, in high EPT matches, players expended more energy and had fewer opportunities to regain physical capacity [14, 15]. In addition, several studies explored how PP could affect match performance, and they found that high-possession teams exhibit lower MRP compared to low-possession teams [11, 13, 18], and researchers also suggested that prolonged possession might reduce match intensity, particularly in high-speed running [14, 16].

Regarding different playing positions, Altmann, Forcher [16] reported that the MRP of players can be influenced significantly by playing positions, which can be in line with the findings of this study. This study found that players’ MRP during each PS can be affected differently by PP according to their playing positions (Figure 3). Moreover, the model results can also provide further information about MRP during each PS for teams with different PP. For teams with high PP (> 50%), both EM and F can have greater HID during IP than OP, but their HID during IP would become lower than OP when PP is over 69% which is extremely high PP in soccer matches. Highpossession teams may employ high pressing during IP and pass the ball to create scoring opportunities in the attacking half, which reduces the space available for wingers and forwards to run at high speed or sprint [13]. On the other hand, if they lose possession, forwards and wingers are expected to press the opponents to prevent a counterattack. As a result, their running work rate during IP decreases, but their MRP during OP increases. Interestingly, almost no teams would have PP lower than 10%, and hence, CD’s and CM’s HID during OP can be greater than IP for most matches. With regards to TD, teams with high PP (> 50%), their CD, ED, CM, and EM performed greater TD during OP than IP, and players with all positions would have the same situation when PP is over 61.8%.

These interesting findings revealed the different effects of PP on different positional players’ MRP, and can provide further evidence for previous findings [4, 11, 12, 13, 18]. For instance, when teams play in low PP, they typically organize defensive activities for the majority of the match and attempt to use counterattacks to score goals, which consumes a large amount of physical energy of players [13]. In such scenarios, it is often the wide players (ED and EM) and F who make runs from the backwards pitch to the attacking third, thereby creating opportunities for counterattacks [31]. Furthermore, as EM and F are required to continuously move during attacking phases to create space and attacking opportunities, players in these positions tend to exhibit higher MRP during IP compared to OP, even when their team have a higher level of PP [32, 33, 34]. In the other hand, although high-PP teams maintain the ball for the majority of the match, which could lead to less fatigue, defending against the opponent’s counterattacks requires defenders to rush back and rebuild the defensive line quickly, and as a result, defensive players tend to exhibit higher MRP during OP compared to IP [13, 24]. However, different defensive strategies may affect this trend, as tactical objectives influence defenders’ approaches, such as high-pressing or lowblock defending.

### Difference between the model and the t-test

The results of this study revealed that there are multiple variables that can affect MRP in soccer matches, and the t-test only considered the main variable, PS. As the results of the t-test, players’ MRP can always be lower during IP than OP, whereas the model suggested the relative levels of MRP under different PS vary depending on the team’s PP. However, the comparison results of the t-test aligned with the model’s outcomes when PP exceeds a certain threshold. To some extent, the results of the t-test were not inherently different from those of the model. Rather, the t-test results represented the model’s outcomes under specific conditions. To address the reasons underlying this result, it is essential to focus on the interaction between PP and PS. In one related study, Castellano, Errekagorri [5] used PP to divide teams into low-, medium-, and high-possession teams, and compared the MRP for the three kinds of teams. Based on their results, it can be inferred that an increase in PP leads to a decline in MRP during IP and a rise in MRP during OP [5], which is highly similar to the findings of this study.

Previous related studies have not entirely overlooked the influence of PP and PS on MRP in soccer matches. On the one hand, withmatch studies compared MRP between each PS, and consequently reported MRP during OP was higher than IP [5, 12, 35]. On the other hand, between-match studies focused on the possession-level of teams, and investigated the influence of PP on MRP, as reporting high-possession teams showed lower MRP compared with low-possession teams [11, 13, 18]. However, previous studies did not integrate multiple influencing factors but instead performed comparisons based on a single primary factor. That approach may lead to results that represent only a partial picture. Therefore, relying solely on ttests (or comparisons based on samples divided by a primary factor) may not provide an accurate representation of the true situation, as the final results might reflect data from specific conditions only. Even though the models we developed in this study may not be the best, the models that we developed at least, during the exploratory process, can contribute to uncovering part of the complexities hidden beneath the surface.

### Strengths, Limitations, and Future Directions

First and most important, this study is the first to compare the use of linear mixed models with t-tests to further explore methods for evaluating players’ MRP in soccer matches. Additionally, this study aimed to further optimize the calculation methods for performance metrics in soccer matches. It is well known that complexity and variability are inherent characteristics of soccer matches, influenced by multiple contextual factors [9, 19] and various tactical effects on match performance [4, 6, 8, 36, 37]. Thus, relying solely on primary factors to evaluate match performance cannot fully capture the most accurate results. As demonstrated in this study, the results of the t-test merely reflect the model’s outcomes under specific conditions. Regarding future analysis, we suggested integrating multiple influencing factors wherever possible. For instance, in order to minimize data loss during analysis, constructing multivariate linear models or, as pointed out by Memmert, Lemmink [37], nonlinear machine learning approaches may offer a potential solution to address this issue effectively. Methods such as SHAP offer interpretability benefits [38], while neural networks excel at simulating the dynamic nature of soccer [39].

Furthermore, the final model established in this study may not be perfect, as it might have omitted relevant variables and is limited to analyzing data from the Spanish La Liga. Additionally, incorporating more MRP metrics at different speed thresholds, such as high-speed running distance, sprinting distance and accelerations, can help future studies uncover further insights into the interaction effects between PS and PP. Future research could explore additional variables to build more comprehensive models and incorporate data from other national leagues. Ultimately, we hope this study serves as a starting point, paving the way for ongoing exploration to develop improved methodologies for calculating performance metrics in soccer matches.

The findings of this study have several practical applications in soccer performance analysis and training. Coaches and performance analysts can use these results to better quantify the physical demands of players during different phases of play (IP and OP) and under varying ball possession levels. For instance, teams that favour high-pressing and possession-based styles can incorporate more HID training sessions to enhance defenders’ MRP when they lose possession and face an opponent’s counterattack. By understanding the positional differences in MRP, tactical strategies can be optimised to enhance team performance and player efficiency. Additionally, the use of linear mixed models offers a more comprehensive approach, such as interaction effects between factors, for evaluating match performance compared to traditional t-tests, allowing practitioners to account for multiple influencing factors. These insights can also aid in workload management, recovery strategies, and the development of performance metrics that better reflect the complexities of modern soccer.

## CONCLUSIONS

This study employed a multivariable linear mixed model to analyse MRP under different PS and found that MRP varied with PP during each PS (IP and OP). Notably, the study revealed that while MRP during OP is generally higher, the trend can reverse in favour of IP states under certain conditions, particularly for teams with lower PP. Additionally, MRP differed across playing positions, emphasising the diverse physical demands placed on players based on their tactical roles.

Subsequently, the study compared the results of traditional t-tests with those of the mixed model, revealing that the t-test results aligned with the model’s findings only under specific circumstances. Accordingly, this research addressed the two research questions proposed in the first section and highlights the limitations of traditional comparative analysis methods, which fail to capture a more comprehensive and accurate representation of match performance. This approach provided deeper insights into the complex interactions between variables like PS and PP, offering a more nuanced understanding than previous studies relying on simplified comparisons. In conclusion, this research highlighted the need for holistic methodologies in sports performance analysis and provides a foundation for the continued development of metrics that better reflect the complexities of soccer matches.

## Data Availability

The respective data are a property of Laliga and are not publicly available. Therefore, in the first place, the authors do not have permission to share the data publicly

## Code availability statement

The codes used for the statistical analysis presented in this paper will be made available upon request to the corresponding author.
